# Diabetes care and outcomes of pediatric refugees and migrants from Ukraine and Syria/Afghanistan with type 1 diabetes in German-speaking countries

**DOI:** 10.3389/fendo.2024.1403684

**Published:** 2024-06-07

**Authors:** Marie Auzanneau, Christina Reinauer, Julian Ziegler, Sven Golembowski, Carine de Beaufort, Hannah Schöttler, Eva Hahn, Joaquina Mirza, Angela Galler, Michael Wurm, Reinhard W. Holl

**Affiliations:** ^1^ Institute of Epidemiology and Medical Biometry, Ulm University, Ulm, Germany; ^2^ German Center for Diabetes Research (DZD), Neuherberg, Germany; ^3^ Department of General Pediatrics, Neonatology and Pediatric Cardiology, Medical Faculty and University Hospital Düsseldorf, Heinrich Heine University, Düsseldorf, Germany; ^4^ Pediatric Diabetology, University Children’s Hospital Tübingen, Tübingen, Germany; ^5^ Department of Pediatrics, Sana Hospital Lichtenberg, Berlin, Germany; ^6^ Department of Pediatric Diabetes and Endocrinology, Centre Hospitalier de Luxembourg, Luxembourg, Luxembourg; ^7^ Faculty of Science, Technology and Medicine, University of Luxembourg, Esch-Belval, Luxembourg; ^8^ Diabetology and Endocrinology, Darmstädter Kinderkliniken Prinzessin Margaret, Darmstadt, Germany; ^9^ Department of Pediatrics and Adolescent Medicine, St. Agnes-Hospital Bocholt – Klinikum Westmünsterland, Bocholt, Germany; ^10^ Kinderkrankenhaus Amsterdamer Straße, Klinik für Kinder- und Jugendmedizin, Paediatric Diabetology, Kliniken Köln, Köln, Germany; ^11^ Charité - Universitätsmedizin Berlin, Sozialpädiatrisches Zentrum, Berlin, Germany; ^12^ Department of Pediatrics, Klinik St. Hedwig, University Hospital Regensburg, Krankenhaus Barmherzige Brüder, Regensburg, Germany

**Keywords:** type 1 diabetes, children, migration, refugees, HbA1c

## Abstract

**Introduction:**

Currently, over two million war refugees live in Germany. Exposure to war and flight is associated with a high burden of diseases, not limited to mental disorders and infections. We aimed to analyze diabetes treatment and outcomes of pediatric refugees and migrants from Ukraine and Syria/Afghanistan with type 1 diabetes (T1D) in German-speaking countries.

**Materials and methods:**

We included patients with T1D documented between January 2013 and June 2023 in the German/Austrian/Luxembourgian/Swiss DPV registry, aged < 20 years, born in Ukraine [U], in Syria or Afghanistan [S/A], or without migration background [C]. Using logistic, linear, and negative binomial regression models, we compared diabetes technology use, BMI-SDS, HbA1c values, as well as severe hypoglycemia and DKA rates between groups in the first year of treatment in the host country. Results were adjusted for sex, age, diabetes duration, and time spent in the host country.

**Results:**

Among all patients with T1D aged < 20 years, 615 were born in Ukraine [U], 624 in Syria or Afghanistan [S/A], and 28,106 had no migration background [C]. Compared to the two other groups, patients from Syria or Afghanistan had a higher adjusted BMI-SDS (0.34 [95%-CI: 0.21–0.48] [S/A] vs. 0.13 [- 0.02–0.27] [U] and 0.20 [0.19–0.21] [C]; all p<0.001), a lower use of CGM or AID system (57.6% and 4.6%, respectively [S/A] vs. 83.7% and 7.8% [U], and 87.7% and 21.8% [C], all p<0.05) and a higher rate of severe hypoglycemia (15.3/100 PY [S/A] vs. 7.6/100 PY [C], and vs. 4.8/100 PY [U], all p<0.05). Compared to the two other groups, patients from Ukraine had a lower adjusted HbA1c (6.96% [95%-CI: 6.77–7.14] [U] vs. 7.49% [7.32–7.66] [S/A] and 7.37% [7.36–7.39] [C], all p<0.001).

**Discussion:**

In their first treatment year in the host country, young Syrian or Afghan refugees had higher BMI-SDS, lower use of diabetes technology, higher HbA1c, and a higher rate of severe hypoglycemia compared to young Ukrainian refugees. Diabetologists should be aware of the different cultural and socioeconomic backgrounds of refugees to adapt diabetes treatment and education to specific needs.

## 1 Introduction

Since the Russian invasion in February 2022, more than six million people fled Ukraine (1). Most of those forcibly displaced individuals moved to other European countries. Germany and Poland accommodated the highest number of Ukrainian refugees (2, 3). At the beginning of 2024, more than one million Ukrainian citizens lived in German-speaking countries. Germany is also the host country of about 400,000 war refugees from Afghanistan and more than 800,000 refugees from Syria, who came since the beginning of the civil war in 2011 (2). Among these refugees, more than one-third are children (2). Pediatric war refugees are a particularly vulnerable group in terms of health. Exposed to the lack of vaccinations, medical exams, and affordable medication in their home country due to the war, confronted with adverse experiences before and during the flight, they have an increased risk of psychological disorders, infections, and poor health outcomes due to insufficient management of chronic conditions.

Data about the specific situation of pediatric refugees or migrants from war zones with type 1 diabetes (T1D) are very scarce. A recent study analyzed the impact of continuous glucose monitoring (CGM) on glycemia in Ukrainian children and adolescents in Czechia (4). However, this study did not report the level of diabetes care and outcomes of these refugees compared to other children and adolescents with T1D.

Using real-world data from a large prospective German/Austrian/Luxembourgian/Swiss diabetes registry, we examined whether diabetes treatment (especially the use of diabetes technology) and outcomes differ between pediatric refugees/migrants with T1D from Ukraine, pediatric refugees/migrants with T1D from Syria or Afghanistan, and children with T1D without migration background.

## 2 Materials and methods

### 2.1 Data source and study population

This observational cross-sectional study is based on data from the German/Austrian/Luxembourgian/Swiss diabetes prospective follow-up registry (DPV) (5). Twice a year, more than 500 diabetes centers transmit their locally collected longitudinal benchmarking data in pseudonymized form to Ulm University, Germany. After plausibility checks and correction, the data is aggregated and completely anonymized for medical research. Data analysis has been approved by the ethics committee of the Medical Faculty of Ulm University (ethics approval 314/21). Informed consent to participate in the DPV register was obtained from patients or their guardians, in agreement with the administrators responsible for data protection at each participating center (5, 6).

As of September 2023, the registry comprised 707,060 patients with any type of diabetes living in Germany, Austria, Switzerland, and Luxembourg. Currently, the DPV registry covers an estimated proportion of more than 90% of all pediatric patients with diabetes (6). For this analysis, we included all patients with T1D newly documented between January 2013 and June 2023, aged under 20 years, born in Ukraine [U], in Syria or Afghanistan [S/A], or without migration background (patient and both parents born in Germany, Austria, Switzerland, or Luxembourg) as a control group [C]. The analysis did not include patients from other countries of origin.

### 2.2 Demographic and clinical variables

The definition of T1D is based in the DPV registry on the physician’s diagnosis according to the international guidelines (7). The use of insulin analogs, CGM, insulin pumps, or automated insulin delivery systems (AID, also called “hybrid-closed-loop” systems) was defined as any use in the observation period. Severe hypoglycemia, defined as a seizure or loss of consciousness requiring external assistance for administration of carbohydrates or glucagon, was self-reported and documented by the physician. Incident diabetic ketoacidosis (DKA) was defined as pH <7.3 or serum bicarbonate <15 mmol/L at least 8 days after diagnosis [all grades of DKA, according to ISPAD guidelines 2018 (8)]. HbA1c values were standardized to the Diabetes Control and Complications Trial reference range (4.05–6.05% [21–43 mmol/mol]) to correct for different laboratory methods (9). Standard deviation body mass index scores (BMI-SDS) were given using the KIGGS references of the German Robert Koch Institute (10).

### 2.3 Statistical analysis

We aggregated treatment and outcomes parameters of patients born in Ukraine, Syria or Afghanistan in the first treatment year in the host country as median, and compared with median values of patients without migration background in the time period from 2013 to June 2023. Unadjusted values are presented as median with lower-upper quartiles for continuous variables, as well as absolute numbers and percentages for variables with binomial distribution. Comparison between groups was performed using Wilcoxon test for continuous variables and X^2^ test for variables with binomial distribution, adjusting for multiple comparisons according to the Holm-Bonferroni step-down procedure. Logistic, linear, and negative binomial regression models were performed, with adjustment for sex, age, diabetes duration, and time spent in the host country, to compare treatment modalities (use of CGM, insulin pump, and AID) and outcomes (BMI-SDS, HbA1c, rates of severe hypoglycemia, and DKA) between groups. Results of regression analyses are presented as adjusted proportions (least square means) with 95%-confidence intervals (95%-CI). A p-value < 0.05 in two-sided tests was considered statistically significant. Statistical analyses were conducted using SAS version 9.4 (build TS1M7, SAS Institute Inc, Cary, NC).

## 3 Results

**Figure 1:**
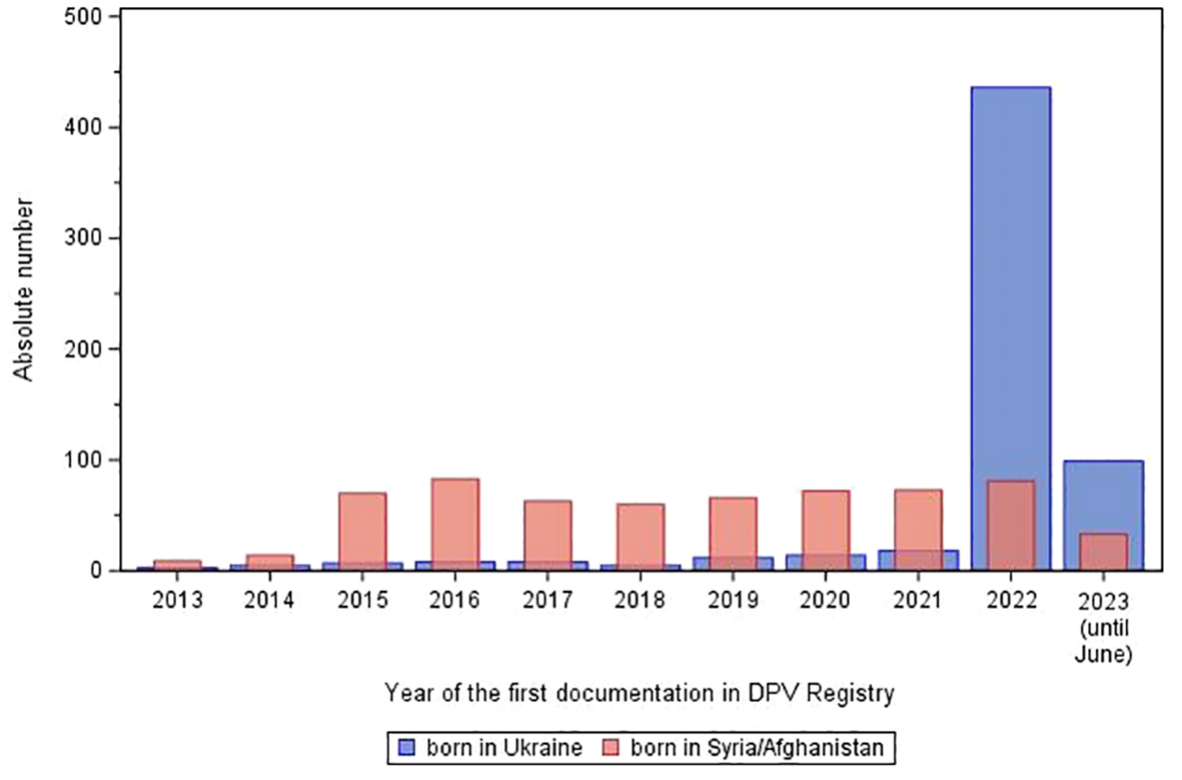
Absolute number of refugees/migrants born in Ukraine or in Syria or Afghanistan aged < 20 years with type 1 diabetes in the DPV Registry by first treatment year in the host country.

Over the last ten years combined, 47,642 children and adolescents with T1D under the age of 20 have been newly documented in the DPV registry. Among these patients, we included 28,106 children and adolescents without migration background [control group, C], 615 born in Ukraine [U], and 624 born in Syria or Afghanistan [S/A]. Of the 615 patients from Ukraine, 535 (87%) arrived since 2022. **Figure 1** represents the absolute number of pediatric war refugees/migrants documented in the DPV registry by country of origin by first treatment year in the host country. In their first treatment year in the host country, most patients born in Ukraine had been living in the host country for less than 1 year, most patients born in Syria/Afghanistan for 1 to 4 years (**Table 1**).

**Table 1:** Characteristics of the study population by migration background in the first treatment year in the host country.

	A. Born in Ukraine (n= 615)	B. Born in Syria/Afghanistan (n= 624)	C. Without migration background* (n= 28,106)	P-values for comparison***		
				A. vs. B.	A. vs. C.	B. vs. C.
Male gender	307 (49.9)	349 (55.9)	12,060 (54.8)	0.241	0.335	1.000
Age, years	11.6 (8.3– 14.3)	11.8 (8.7 – 15.0)	12.0 (8.1 – 14.9)	0.241	0.228	1.000
Diabetes duration, years	2.4 (0.6–5.3)	0.6 (0.3 – 4.2)	1.6 (0.7 – 2.8)	**<0.001**	**<0.001**	**<0.001**
Time spent in the German-speaking country	0 (0 – 1)	1 (1 – 4)	12 (8 – 15)	**<0.001**	**<0.001**	**<0.001**
BMI SDS**	0.02 (-0.59 – 0.63)	0.24 (-0.42 – 0.84)	0.22 (-0.41 – 0.84)	**<0.001**	**<0.001**	1.000
HbA1c, %	7.22 (6.39 – 8.37)	7.59 (6.77 – 8.77)	7.20 (6.58 – 7.90)	**0.003**	0.604	**<0.001**
Insulin dose (/kg)	0.87 (0.68 – 1.13)	0.83 (0.63 – 1.09)	0.74 (0.58 – 0.92)	0.241	**<0.001**	**<0.001**
Use of insulin analogs	597 (97.1)	580 (93.0)	27,561 (98.1)	**0.012**	0.396	**<0.001**
Use of insulin pump	153 (24.9)	124 (19.9)	16,608 (59.1)	0.241	**<0.001**	**<0.001**
Use of CGM	502 (81.6)	340 (54.5)	24,019 (85.5)	**<0.001**	0.070	**<0.001**
Use of AID	54 (8.8)	29 (4.7)	6,861 (24.4)	**0.043**	**<0.001**	**<0.001**

Unadjusted values are given as median (lower-upper quartiles) for continuous variables and absolute number (percentages) for binary variables.

*For patients without migration background, the data have been aggregated as median of the observation period January 2013 – June 2023.

**BMI SDS, standard deviation score of Body Mass Index (kg/m2) using KIGGS (RKI) reference data.

***Comparison using Wilcoxon test for continuous variables and X^2^ test for variables with binomial distribution, adjusted for multiple comparisons according to the Holm-Bonferroni stepdown procedure. P<0.05 (two-sided) was considered statistically significant (in bold).

### 3.1 Comparison of the three groups of patients

Descriptive data for each group are presented in **Table 1**. In their first treatment year in the host country, age did not differ significantly between the two groups of refugees and was similar to the median age of the children and adolescents without migration background in the whole time period (median age: 11.6 years [U] vs. 11.8 years [S/A], and 12.0 years [C]). Patients from Ukraine had a lower BMI-SDS (median: 0.02 [U] vs. 0.24 [S/A], and 0.22 [C]), as well as the longest diabetes duration (median: 2.4 years [U] vs. 0.6 years [S/A] and 1.6 years [C]). Patients from Syria/Afghanistan had the lowest use of insulin analogs (median: 93% [S/A] vs. 97% [U] and 98% [C]), the lowest use of CGM and AID (median: 55% and 5%, respectively [S/A] vs. 82% and 9% [U] and 86% and 24% [C]) and the highest HbA1c value (median: 7.59% [S/A] vs. 7.22% [U] and 7.20% [C]). P-values for comparison between groups are indicated in **Table 1**.

### 3.2 Comparison by location of the diabetes diagnosis

The majority of the young refugees from Ukraine (n=488/615, 79.3%) had their diabetes diagnosis in their home country, whereas most young refugees from Syria/Afghanistan were diagnosed after they arrived in the host country (n=336/624, 53.8%). Patients from Ukraine diagnosed in their home country were younger, a lower HbA1c, a lower BMI-SDS and used more CGM, insulin pumps and AID than patients from Syria/Afghanistan diagnosed in their home country (median age: 11.6 vs. 12.9 years; BMI-SDS: -0.02 vs. 0.27; HbA1c: 7.37% vs. 8.18%; CGM: 83.4% vs. 39.9%; pump: 26.8% vs. 13.9%; AID: 9.0% vs. 2.4%; all p<0.001). In contrast, in patients diagnosed after arrival in the host country, no significant differences in these parameters could be observed (median age: 11.1 vs. 11.1 years; BMI-SDS: 0.08 vs. 0.19; HbA1c: 6.83% vs. 7.04%; CGM: 74.8% vs. 67.0%; Pump: 17.3% vs. 25.0%; AID: 7.9% vs. 6.6%; all p=1.00).

### 3.3 Adjusted analyses

**Figure 2:**
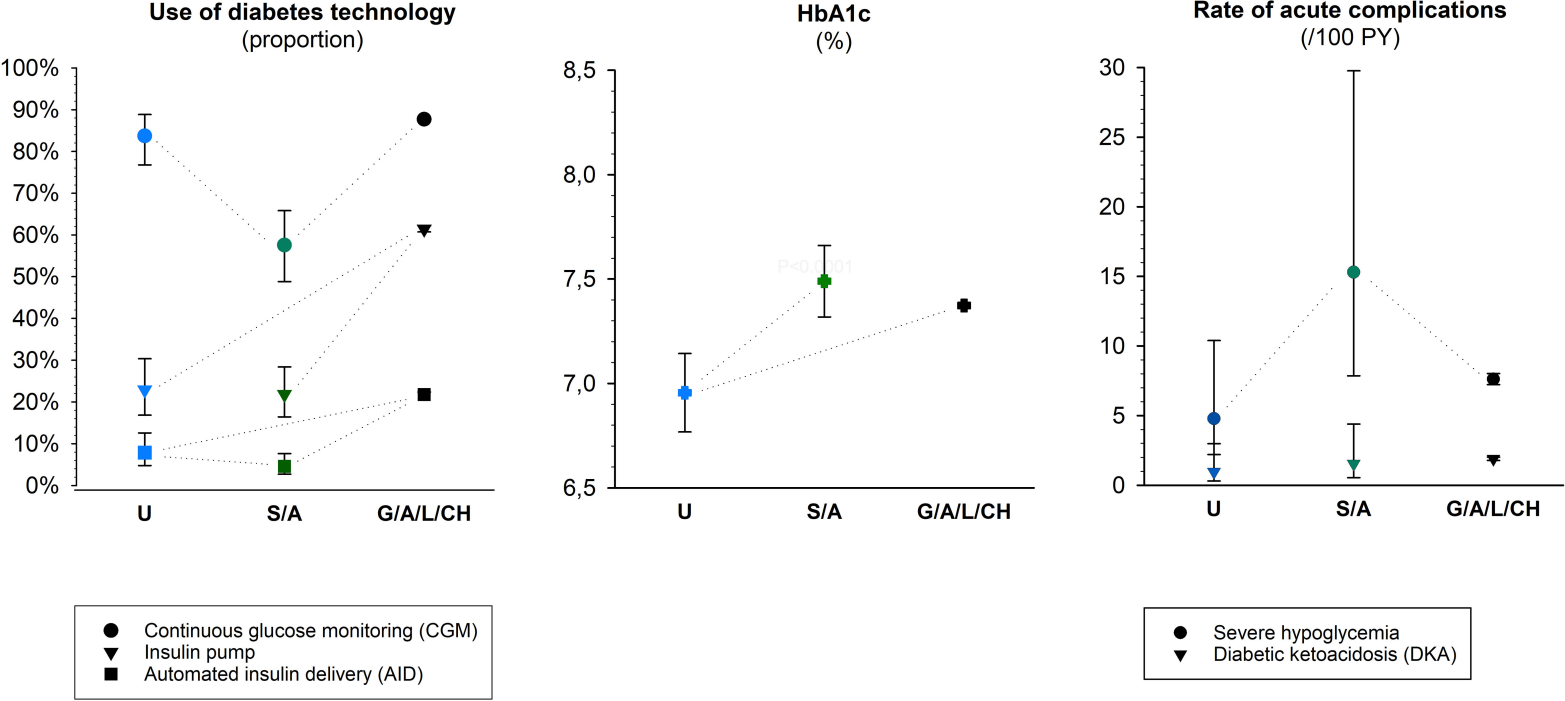
Diabetes treatment and outcomes in war refugees/migrants aged < 20 years with type 1 diabetes compared in the first treatment year in the host country with peers without migration background. Represented are the estimates (Least-Square-means) with 95%-confidence intervals for the three origin groups from logistic (use of CGM, insulin pump, and AID), linear (HbA1c), and negative binomial (rates of severe hypoglycemia and DKA) regression models, adjusted for sex, age, diabetes duration, and time spent in the host country. The dotted lines indicate the significant comparisons (p<0.05).

After adjustment for sex, age, and diabetes duration, and time spent in the host country, the BMI-SDS was significantly higher in patients from Syria/Afghanistan compared to other groups (0.34 [95%-CI: 0.21–0.48] [S/A] vs. 0.13 [- 0.02–0.27] [U] and 0.20 [0.19–0.21] [C]). Other results of the adjusted regression models are shown in **Figure 2**. Patients from Syria/Afghanistan less frequently used a CGM or an AID system than both other groups (CGM and AID, respectively: 57.6% and 4.6% [S/A] vs. 83.7% and 7.8% [U], and vs. 87.7% and 21.8% [C], all p<0.05). Both groups of refugees less frequently used an insulin pump compared to patients without migration background (pump: 21.8% [S/A] and 22.9% [U] vs. 61.5% [C]; both p<0.001). Mean HbA1c values were significantly lower in patients from Ukraine compared to the other groups (6.96% [95%-CI: 6.77–7.14] [U] vs. 7.49% [7.32–7.66] [S/A] and 7.37% [7.36–7.39] [C]; both p<0.001). The rate of severe hypoglycemia was significantly higher in patients from Syria/Afghanistan compared to the two other groups (15.3/100 PY [S/A] vs. 7.6/100 PY [C], p<0.001, and vs. 4.8/100 PY [U]; p=0.047). The differences in DKA rates were not significant.

## 4 Discussion

This observational multicenter study describes diabetes treatment and outcomes of young refugees with T1D in their first treatment year in a German-speaking country and points out differences depending on the country of origin. After multiple adjustments, we found that young refugees from Syria or Afghanistan had higher BMI-SDS, lower use of CGM or AID system, higher adjusted HbA1c, and higher rate of severe hypoglycemia than young refugees from Ukraine.

A variety of reasons, not just war, may have led refugees to leave their home country over the period analyzed (2013–2023). In particular, this analysis includes 80 Ukrainian children who moved before the Russian invasion in February 2022. Beyond war, the aim of this study was to compare migrants by region of origin over the same period, to reflect the possible impact of cultural and socio-economic differences on diabetes outcomes.

The use of insulin pump or AID systems was significantly less frequent in both groups of refugees than in patients without migration background, and the use of CGM was far less frequent in refugees from Syria/Afghanistan than in the two other groups. For both groups of refugees, we compared the first treatment year in the host country, which was in median 2018-2019 for children from Syria or Afghanistan and 2022 for children from Ukraine. Overall, the use of diabetes technology, and especially of CGM, increased between these years and this evolution partly explain the higher use of CGM in Ukrainian children. Anyway, the much higher use of CGM in Ukrainian children in their first treatment year in the host country may contribute to lower HbA1c in comparison to Syrian or Afghan children (4). In Germany, Austria, Switzerland, and Luxembourg, all refugees benefit from a special medical assistance covering care for chronic diseases such as diabetes. Access to diabetes technology for young people with migration background in Germany has improved over the past decade, but significant disparities persist (11, 12). Therefore, it is crucial to promote the use of diabetes technology and most particularly of CGM, since these devices have been proven to improve diabetes control in youth (4, 6, 13).

Differences in age, BMI, HbA1c, and use of diabetes technology were not significant when refugees were diagnosed in the host country, but refugees diagnosed in Syria/Afghanistan had significantly higher HbA1c values than refugees diagnosed in Ukraine. This worse glycemic control may be associated with the lower use of diabetes technology and with the poorer quality of diabetes care in Syria/Afghanistan compared to Ukraine. About half the young refugees from Syria/Afghanistan were first treated in their home country before coming to a German-speaking country, and for most of them, diabetes outcomes are likely to last after arrival, because patients and their parents need time and appropriate education to change habits in diabetes management.

Several publications pointed out cultural or socioeconomic characteristics leading to differences in the health status of refugees depending on their country of origin (14–16). Dietary habits, for example, vary by region of origin and are associated with T1D outcomes. Even after adjusting for demographics and time spent in the host country, refugees from Syria/Afghanistan had a higher BMI-SDS than the two other groups. At least for healthy adults, there is evidence of inadequate nutritional intake among Syrian refugees in Germany (15, 17). Moreover, in accordance with our findings, studies have reported that the prevalence of overweight, including obesity, was higher in pediatric refugees from Syria (15) or refugees from Afghanistan (16) compared to peers without a migration background. Another aspect is that German dietary education programs reflect European eating habits, which are closer to Ukrainian eating habits than to Syrian or Afghan ones. Dietary education for this last group of refugees in German-speaking countries is therefore more difficult.

Education level and more specifically health literacy is also known to play an important role in T1D outcomes. A review of the current literature highlights the high education level of the Ukrainian refugees: more than two-thirds of them hold a tertiary/university educational degree (3). This was only the case for 11% of the Syrian refugees and 21% of the refugees from Afghanistan living in Germany in 2022 (18). Moreover, about half of the Ukrainian refugees report to speak English (3). Language skills are not only crucial for inclusion in the host country, but also for adequate communication with diabetes teams and effective participation in diabetes education programs. Knowledge of English is particularly useful, as good quality English translations are widely available in German-speaking countries. These differences can also contribute to explain the better glycemic control of the Ukrainian refugees compared to peers from Syria or Afghanistan in this analysis.

Another factor that should be taken into account is mental health. Mental comorbidities may contribute to worse glycemic control. A high prevalence of psychological distress, including depression and posttraumatic disorders due to war and flight in a foreign country has been reported in Syrian and Afghan refugees (16, 19). Acute and long-term psychological distress may still be underreported among Ukrainian refugees who have just recently moved to the host country.

### 4.1 Strengths and limitations

The use of the large multicenter DPV registry, which covers 93% of the pediatric population with T1D in Germany (20), is the main strength of this study. Our data collected over ten years is expected to comprise almost all children and adolescents with T1D who migrated to Germany from Syria/Afghanistan and Ukraine. The main limitation of this study is that we did not include several important factors, like food intake, socioeconomic situation of the child or the parents (education/school, income, occupation/work), family situation (child living with at least one parent or unaccompanied), or type of accommodation (home or refugee center), because these data were not available in the registry. Moreover, we did not include mental comorbidities, as they are not part of the standardized documentation. As described above, such information would be important for a better understanding of the differences in T1D outcomes. However, these factors can be considered mediators (intermediate factors on the causal pathway) and not confounders in the relationship between the country of origin and the diabetes outcomes. In fact, they are influenced by the country of origin but there is no inverse relationship. Therefore, the association between country of origin and outcomes must be valid without adjustment for these factors. A further limitation is that BMI-SDS values were calculated based on German references since specific references for Ukraine, Syria, or Afghanistan were unavailable. Lastly, this cross-sectional study provides an overview of diabetes care disparities in pediatric refugees but does not provide information about changes over time. Therefore, longitudinal studies are needed in the future to analyze the impact of diabetes care interventions for refugees over time.

## 5 Conclusion

We aimed to assess whether young refugees with T1D present specific characteristics depending on their country of origin. This knowledge is useful for adapting diabetes care and offering the best possible management in the host countries. We found that in their first treatment year in the host country, young refugees from Syria or Afghanistan had a higher BMI-SDS, a lower use of CGM or AID system, a higher adjusted HbA1c, and a higher rate of severe hypoglycemia than young refugees from Ukraine. The different years of arrival in the host country, but also socioeconomic and cultural aspects, in particular language barriers or dietary education based on European habits can explain some of the differences between refugees from Syria or Afghanistan and those from Ukraine. Healthcare providers and diabetologists should be aware of these differences between refugees with T1D, depending on their country of origin, to be able to adapt treatment and diabetes education to specific cultures and needs (21). For optimal diabetes control, culturally sensitive education and communication is necessary. It should take into account eating habits from the home country, language knowledge, and health literacy.

## Data availability statement

The original contributions presented in the study are included in the /supplementary files. Further inquiries can be directed to the corresponding authors.

## Ethics statement

The studies involving humans were approved by the ethics committee of the Medical Faculty of Ulm University (ethics approval 314/21). The studies were conducted in accordance with the local legislation and institutional requirements. Written informed consent for participation in this study was provided by the participants’ legal guardians/next of kin.

## Author contributions

MA: Conceptualization, Formal analysis, Methodology, Software, Writing – original draft, Writing – review & editing. CR: Writing – review & editing. JZ: Writing – review & editing. SG: Writing – review & editing. CD: Writing – review & editing. HS: Writing – review & editing. EH: Writing – review & editing. JM: Writing – review & editing. AG: Writing – review & editing. MW: Writing – review & editing. RH: Conceptualization, Data curation, Funding acquisition, Writing – review & editing.

## References

[1] United Nations High Commissioner for Refugees (UNHCR). Ukraine refugee situation. Available online at: https://data.unhcr.org/en/situations/Ukraine/location?secret=unhcrrestricted.

[2] Mediendienst integration. Available online at: https://mediendienst-integration.de/migration/flucht-asyl/ukrainische-fluechtlinge.html.

[3] Kohlenberger J Buber-Ennser I Pędziwiatr K Rengs B Setz I Brzozowski J. High self-selection of Ukrainian refugees into Europe: Evidence from Kraków and Vienna. PloS One. (2023) 18:e0279783. doi: 10.1371/journal.pone.0279783 38117699 PMC10732457

[4] Neuman V Vavra D Drnkova L Pruhova S Plachy L Kolouskova S. Introduction of continuous glucose monitoring (CGM) is a key factor in decreasing HbA1c in war refugee children with type 1 diabetes. Diabetes Res Clin Pract. (2024) 208:111118. doi: 10.1016/j.diabres.2024.111118 38309536

[5] Hofer SE Schwandt A Holl RW Austrian/German DPV Initiative. Standardized documentation in pediatric diabetology: experience from Austria and Germany. J Diabetes Sci Technol. (2016) 10:1042–9. doi: 10.1177/1932296816658057 PMC5032966 27381028

[6] Karges B Tittel SR Bey A Freiberg C Klinkert C Kordonouri O. Continuous glucose monitoring versus blood glucose monitoring for risk of severe hypoglycaemia and diabetic ketoacidosis in children, adolescents, and young adults with type 1 diabetes: a population-based study. Lancet Diabetes Endocrinol. (2023) 11:314–23. doi: 10.1016/S2213-8587(23)00061-X 37004710

[7] Libman I Haynes A Lyons S Pradeep P Rwagasor E Tung JY. ISPAD Clinical Practice Consensus Guidelines 2022: Definition, epidemiology, and classification of diabetes in children and adolescents. Pediatr Diabetes. (2022) 23:1160–74. doi: 10.1111/pedi.13454 36537527

[8] Wolfsdorf JI Glaser N Agus M Fritsch M Hanas R Rewers A. ISPAD Clinical Practice Consensus Guidelines 2018: Diabetic ketoacidosis and the hyperglycemic hyperosmolar state. Pediatr Diabetes. (2018) 19:155–77. doi: 10.1111/pedi.12701 29900641

[9] Diabetes Control and Complications Trial Research Group Nathan DM Genuth S Lachin J Cleary P Crofford O. The effect of intensive treatment of diabetes on the development and progression of long-term complications in insulin-dependent diabetes mellitus. N Engl J Med. (1993) 329:977–86. doi: 10.1056/NEJM199309303291401 8366922

[10] Kurth B-M Kamtsiuris P Hölling H Schlaud M Dölle R Ellert U. The challenge of comprehensively mapping children’s health in a nation-wide health survey: Design of the German KiGGS-Study. BMC Public Health. (2008) 8:196. doi: 10.1186/1471-2458-8-196 18533019 PMC2442072

[11] Icks A Razum O Rosenbauer J Bächle C Hungele A Mönkemöller K. Lower frequency of insulin pump treatment in children and adolescents of turkish background with type 1 diabetes: analysis of 21,497 patients in Germany. Diabetes Technol Ther. (2012) 14:1105–9. doi: 10.1089/dia.2012.0138 PMC3521139 23216338

[12] Auzanneau M Rosenbauer J Maier W Von Sengbusch S Hamann J Kapellen T. Heterogeneity of access to diabetes technology depending on area deprivation and demographics between 2016 and 2019 in Germany. J Diabetes Sci Technol. (2021) 15:1059–68. doi: 10.1177/19322968211028608 PMC8442190 34253084

[13] Lin T Manfredo JA Illesca N Abiola K Hwang N Salsberg S. Improving continuous glucose monitoring uptake in underserved youth with type 1 diabetes: the IMPACT study. Diabetes Technol Ther. (2023) 25:13–9. doi: 10.1089/dia.2022.0347 36223197

[14] Binkowski S Roberts A Fried L Nicholas JA Frearson K Davis EA. Perspectives of culturally and linguistically diverse families in the management of children with type 1 diabetes in Western Australia. Ethn Health. (2023) 28:822–35. doi: 10.1080/13557858.2023.2190063 36935189

[15] Al Masri F Müller M Straka D Hahn A Schuchardt JP. Nutritional and health status of adult Syrian refugees in the early years of asylum in Germany: a cross-sectional pilot study. BMC Public Health. (2022) 22:2217. doi: 10.1186/s12889-022-14684-7 36447164 PMC9706931

[16] Matsangos M Ziaka L Exadaktylos AK Klukowska-Rötzler J Ziaka M. Health status of afghan refugees in europe: policy and practice implications for an optimised healthcare. Int J Environ Res Public Health. (2022) 19:9157. doi: 10.3390/ijerph19159157 35954518 PMC9368211

[17] Sauter A Kikhia S Von Sommoggy J Loss J. Factors influencing the nutritional behavior of Syrian migrants in Germany — results of a qualitative study. BMC Public Health. (2021) 21:1334. doi: 10.1186/s12889-021-11268-9 34229649 PMC8262055

[18] Heß B. Potential of asylum applicants: Analysis of "Social Component" data relating to applicants' social structure. Annual Report 2022 (Reports on Migration and Integration, Series 3). Nuremberg. Federal Office for Migration and Refugees. (2024). doi: 10.48570/bamf.fz.bericht.r3.en.2024.soko.jb.2022.1.0

[19] Kurt G Ventevogel P Ekhtiari M Ilkkursun Z Erşahin M Akbiyik N. Estimated prevalence rates and risk factors for common mental health problems among Syrian and Afghan refugees in Türkiye. BJPsych Open. (2022) 8:e167. doi: 10.1192/bjo.2022.573 36106400 PMC9534906

[20] Stahl-Pehe A Kamrath C Prinz N Kapellen T Menzel U Kordonouri O. Prevalence of type 1 and type 2 diabetes in children and adolescents in Germany from 2002 to 2020: A study based on electronic health record data from the DPV registry. J Diabetes. (2022) 14:840–50. doi: 10.1111/1753-0407.13339 PMC9789390 36515004

[21] Moore TH Dawson S Wheeler J Hamilton-Shield J Barrett TG Redwood S. Views of children with diabetes from underserved communities, and their families on diabetes, glycaemic control and healthcare provision: A qualitative evidence synthesis. Diabetes Med. (2023) 40:e15197. doi: 10.1111/dme.15197 37573564

